# Neutrophil-rich, noncollagenous 16A domain-negative bullous pemphigoid associated with psoriasis

**DOI:** 10.1016/j.jdcr.2021.08.025

**Published:** 2021-09-01

**Authors:** Morgan E. Sussman, Shoshana K. Grossman, Sylvia Hsu, Jason B. Lee, Kiran Motaparthi

**Affiliations:** a Department of Dermatology, Lewis Katz School of Medicine at Temple University, Philadelphia, Pennsylvania; b Department of Dermatology and Cutaneous Biology, Thomas Jefferson University, Philadelphia, Pennsylvania; c Department of Dermatology, University of Florida School of Medicine, Gainesville, Florida

**Keywords:** bullous pemphigoid, NC16A domain, neutrophils, psoriasis, BP, bullous pemphigoid, ELISA, enzyme-linked immunosorbent assay, Ig, immunoglobulin, NC16A, noncollagenous 16A, DIF, direct immunofluorescence

## Introduction

Bullous pemphigoid (BP) is the most common autoimmune bullous disease.^1^ The association between BP and psoriasis has been frequently reported.^2^ The most common histopathologic and serologic findings in patients with BP include a subepidermal blister with eosinophils^3^^,^^4^ and BP180-noncollagenous 16A (NC16A) reactivity, demonstrated using enzyme-linked immunosorbent assay (ELISA).^1^ Uncommonly, the histopathology of BP may be rich in neutrophils. Additionally, epitope spreading, which leads to the diversification of autoantigen targeting, has a role in the production of non-NC16A autoantibodies in patients with BP.^5^ Herein, we present a case of BP associated with psoriasis that demonstrated neutrophilic infiltrates and NC16A domain nonreactivity.

## Case report

**Fig 1:**
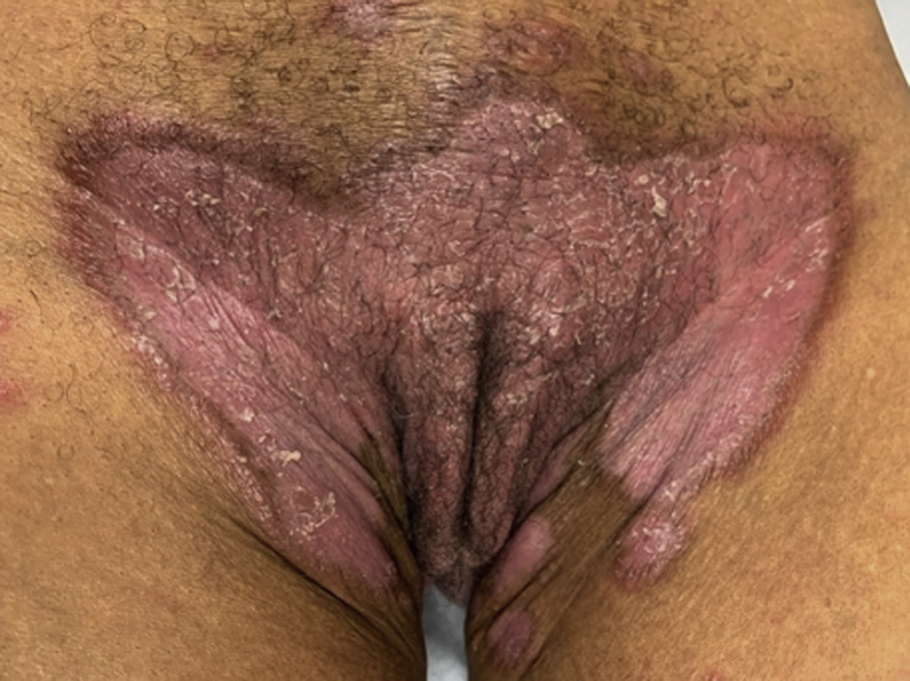
The image shows psoriasis in the patient, with scaly, erythematous plaque in the suprapubic area.

**Fig 2:**
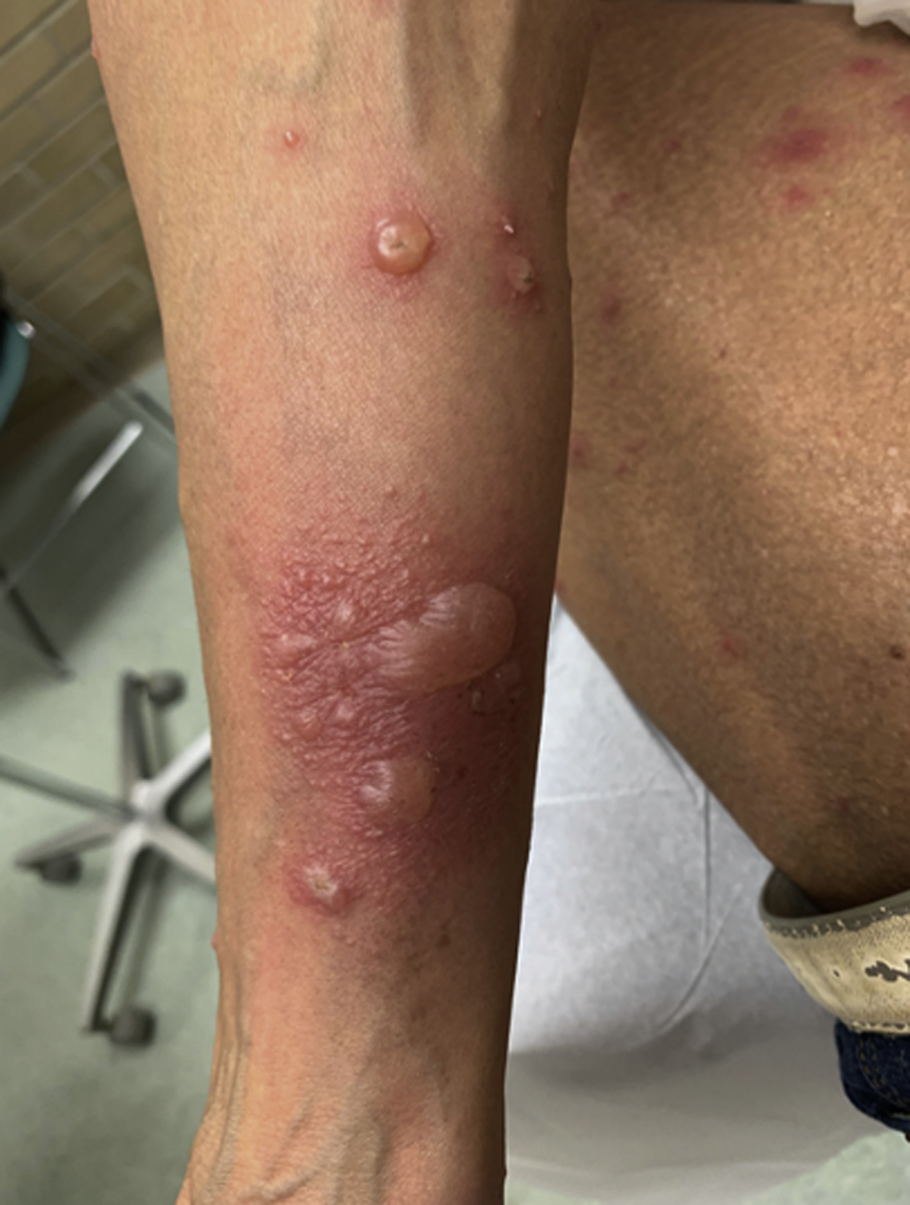
The image shows bullous pemphigoid. Tense bullae can be seen, with surrounding erythema on the upper portion of the right arm.

A 63-year-old woman with an 8-month history of untreated, predominantly inverse psoriasis consisting of erythematous, scaly plaques of the inframammary folds, extensor surfaces, and groin presented with new pruritic blisters on the arms, legs, and trunk (Figs 1 and 2). Mucosal lesions were absent. Based on the clinical features and distribution of the lesions, a clinical diagnosis of coexisting inverse psoriasis and BP was made. Biopsies were performed for routine histopathology and direct immunofluorescence (DIF).

**Fig 3:**
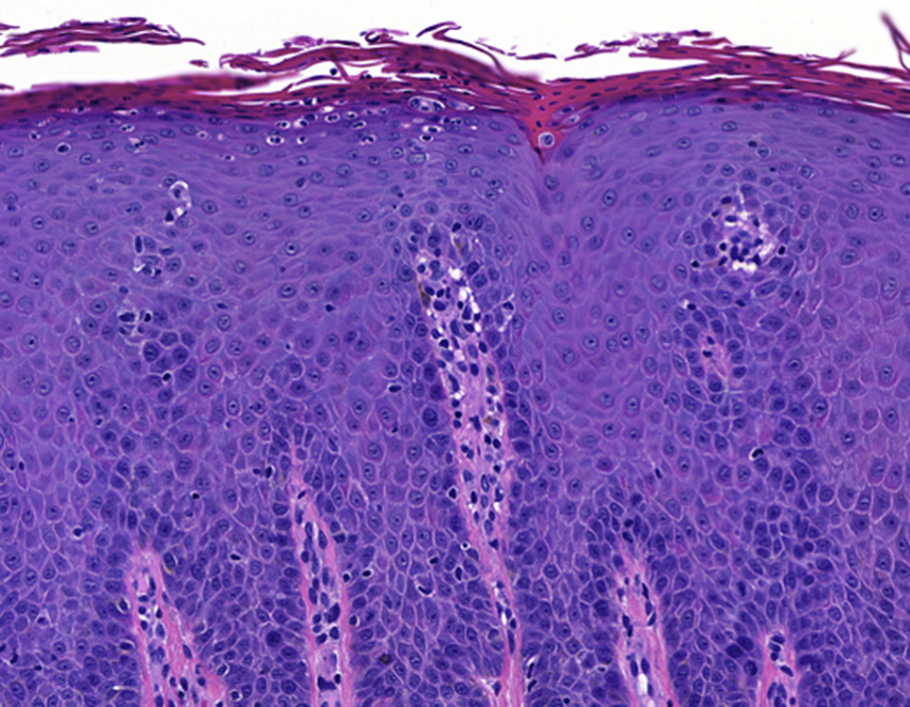
The image shows psoriasis histopathology. Left inframammary fold biopsy: psoriasiform hyperplasia, parakeratosis, absent granular layer, and intracorneal and intraepidermal neutrophils (hematoxylin-eosin stain; original magnification: ×100.)

**Fig 4:**
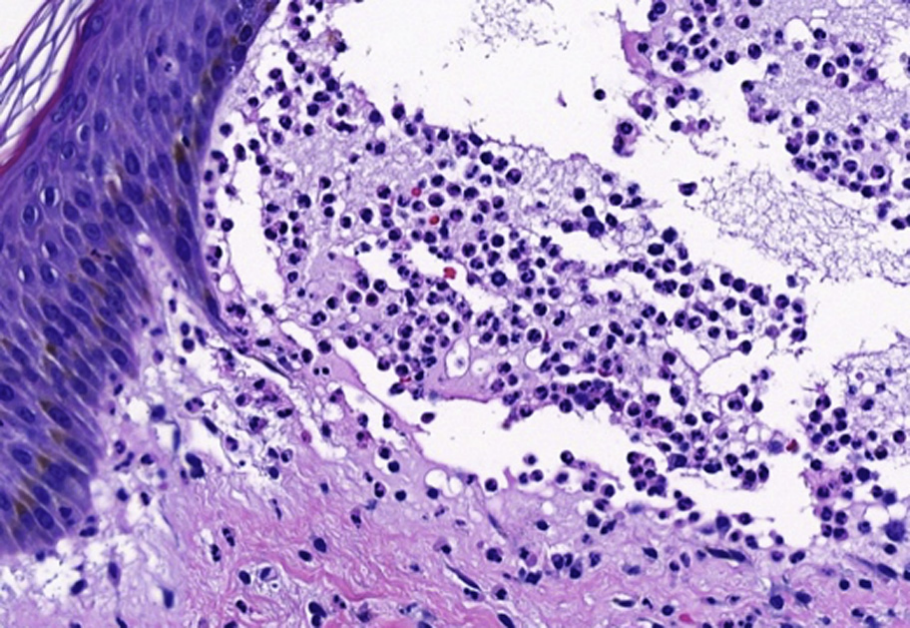
The image shows bullous pemphigoid histopathology. Biopsy of the upper portion of the right arm: subepidermal blister with neutrophils (hematoxylin-eosin stain; original magnification: ×100.)

The histopathology of a plaque obtained from the inframammary fold was diagnostic for psoriasis (Fig 3). The histopathology of a bulla obtained from the upper portion of the arm demonstrated a neutrophil-rich subepidermal bulla, for which the histopathologic differential diagnosis included bullous systemic lupus erythematosus, linear immunoglobulin (Ig) A bullous dermatosis, dermatitis herpetiformis, anti-p200 (anti-laminin gamma 1) pemphigoid, and neutrophil-rich forms of BP and epidermolysis bullosa acquisita (Fig 4). The result of DIF performed on the biopsy material obtained from the right thigh was initially negative. However, repeat DIF performed on another sample obtained from the left thigh demonstrated strong linear deposition of IgG and C3 at the dermoepidermal junction, consistent with pemphigoid. Indirect immunofluorescence with the IgG class using the salt-split skin technique demonstrated immunofluorescence at the basement membrane zone in an epidermal pattern. The titer of antinuclear antibody was only 1:80 in a speckled pattern, and the patient lacked clinical findings of systemic lupus erythematosus. Additionally, the result of ELISA for autoantibodies against type VII collagen was negative. Taken together with the results of indirect immunofluorescence, the diagnoses of epidermolysis bullosa acquisita and bullous systemic lupus erythematosus were less likely. The results of initial and repeat ELISAs for circulating autoantibodies directed against BP180 and BP230 were negative.

Upon initiation of prednisone at 60 mg daily, the patient experienced rapid clinical improvement in both psoriasis and BP. Upon tapering of prednisone, the patient's psoriasis began to flare. At this time, adalimumab was added to the treatment regimen; thereafter, the patient's BP remained under control during the tapering of prednisone.

## Discussion

The decision to repeat DIF was driven by the clinical morphology that was strongly suggestive of BP in association with psoriasis (psoriasis pemphigoides) and by the risk of a false-negative DIF result in 33% of biopsies performed on the lower extremity, although a more recent study demonstrated that the risk of a false-negative DIF result in patients with BP does not vary by anatomic site.^6^ Additionally, a lower DIF positivity rate in patients with comorbid BP and psoriasis than in those with BP alone has been reported.^7^ Patients with psoriasis alone may have a positive DIF result, but this is limited to granular deposition of C5b-9.^4^ Thus, the second DIF in this case, which revealed the linear deposition of IgG and C3, reflected a true-positive result for BP because this pattern is not seen in patients with psoriasis alone.^4^ In this case, the repeat DIF yielded a positive result, whereas ELISA yielded persistently negative results for BP180 and BP230 IgG reactivity. DIF is the gold standard diagnostic test for BP, with a sensitivity of 91%.^8^ In contrast, the sensitivities of BP180 and BP230 ELISAs are 89% and 59%, respectively.^8^^,^^9^

The current literature estimates that about 8%-10% of patients with BP have a negative ELISA result.^1^ This result, however, represents a false-negative result due to the limitations of traditional ELISA testing. Traditional ELISA is used to detect autoantibodies that bind to a specific region of the BP180 structural protein, referred to as the NC16A domain.^1^ In a study by Fairley et al,^1^ up to 7.8% of patients with BP were seronegative for BP180, as determined using ELISA. Additionally, the authors located 4 extracellular antigenic regions outside of the classic NC16A domain. Patients with NC16A domain-negative BP cannot be identified using traditional ELISA.

Epitope spreading is defined as the expansion of immune response to the same antigen, occurring as a result of tissue inflammation or physical proximity.^5^ Epitope spreading occurs within the BP180 protein, leading to the diversification of immune response.^5^ Early in NC16A domain-negative BP, autoantibodies initially target the immunodominant NC16A domain. Chronic tissuse damage then leads to the exposure and recognition of secondary epitopes.^5^ One hypothesis speculates that psoriasis triggers persistent inflammation at the dermoepidermal junction.^2^ It is possible that this chronic inflammation facilitates epitope spreading in patients with NC16A domain-negative BP.

The association between psoriasis and BP has been described in several studies,^2^ and BP is 3.05 times more likely to develop in psoriatic patients than in matched controls. Notably, psoriasis preceded the development of BP in all patients in a case-control study.^10^ Additional reports of NC16A domain-negative BP and concurrent psoriasis have been documented in the literature.^1^^,^^7^ Among a series of patients with BP and comorbid psoriasis, over 25% were seronegative for BP180 NC16A, as determined using ELISA.^7^

Although BP is traditionally associated with an eosinophilic infiltrate,^3^^,^^4^ neutrophil-rich BP has been rarely described.^3^^,^^11^ According to a retrospective review, neutrophils comprised a significant portion of inflammatory infiltrates in 16% of patients.^12^ Neutrophilic predominance has been reported in cases of drug-induced BP,^13^ specifically in association with immune checkpoint inhibitor therapy.^13^ A recent report described neutrophil-rich BP in association with psoriasis.^3^ The authors hypothesized that interleukin 17A, a strong neutrophil chemoattractant that also plays a major role in psoriasis pathogenesis, induces the formation of neutrophil-predominant blisters.^3^

Corticosteroid administration in patients with psoriasis is often avoided because of the potential risk of flares following steroid withdrawal. However, flares of psoriasis occur in less than 2% of patients during or immediately after treatment with systemic corticosteroids.^14^ Although the patient presented here experienced flares following corticosteroid taper, greater evidence supports the use of systemic steroids in patients with BP and psoriasis. Methotrexate or interleukin 17 inhibitors, both of which are effective in both the conditions, can be used concurrently with or after systemic steroids.^15^^,^^16^

## Conflicts of interest

None disclosed.
